# Dr. Edwin R. Maxson

**Published:** 1912-04

**Authors:** 


DR. EDWIN R. MAXSON of Syracuse, a graduate of the University of Syracuse, 1886, died January 25, 1912.

